# Impact of Hoarding and Obsessive–Compulsive Disorder Symptomatology on Quality of Life and Their Interaction With Depression Symptomatology

**DOI:** 10.3389/fpsyg.2022.926048

**Published:** 2022-08-01

**Authors:** Binh K. Nguyen, Jessica J. Zakrzewski, Luis Sordo Vieira, Carol A. Mathews

**Affiliations:** ^1^Department of Psychiatry, College of Medicine, University of Florida, Gainesville, FL, United States; ^2^Center for OCD, Anxiety, and Related Disorders (COARD), University of Florida, Gainesville, FL, United States; ^3^Department of Medicine, College of Medicine, University of Florida, Gainesville, FL, United States

**Keywords:** QoL (quality of life), OCD (obsessive–compulsive disorder), hoarding, depression, mTurk, QoLI (Quality of Life Inventory), psychiatric symptoms, mediation

## Abstract

Hoarding disorder (HD) is a psychiatric condition characterized by difficulty discarding items and accumulation of clutter. Although studies have established the negative impact of HD and compulsive hoarding behavior, fewer have examined the impact on quality of life (QoL) of hoarding behavior independent of obsessive–compulsive disorder (OCD). Moreover, specific aspects of QoL such as success in work/academics or satisfaction with interpersonal relationships have not been well-investigated. In this study, we examined, in a sample of 2100 adult participants obtained from Amazon Mechanical Turk, the relationships between hoarding, OCD, and depression symptomatology and four QoL domains (success, enrichment, environment, and family) derived from a factor analysis of the Quality of Life Inventory (QoLI). We performed linear regressions to examine associations between psychiatric symptomatology and QoL domains and then conducted mediation analyses to investigate the role of depressive symptomatology in the identified relationships. We found that while hoarding and obsessive–compulsive symptoms were both negatively associated with QoL, they were associated with different domains [hoarding was significantly associated (*p* < 0.05) with total QoL and all domains and uniquely associated with environment and family QoL compared to obsessive–compulsive symptoms], whereas obsessive–compulsive symptoms were only significantly associated with total, success, and enrichment QoL. However, when depressive symptoms were included in the model, hoarding no longer accounted for significant variance in the total, environment, or family QoL domains (*p* > 0.05), and was less strongly associated with success or enrichment. Mediation analyses confirmed the role of depression as a complete mediator of hoarding’s effect on total, environment, and family QoL, and as a partial mediator of hoarding’s effect on success and enrichment QoL. Further examination of the relationship between hoarding symptoms and QoL in those with mild, moderate, and severe depression indicated that in those with more severe depression, hoarding was associated with improved QoL, indicating a possible buffering or compensatory effect. The findings suggest a differential impact of hoarding and obsessive–compulsive symptoms on QoL and emphasize the importance of considering co-morbid depressive symptoms in designing more targeted interventions. Future studies should continue to investigate these complex relationships, given the high co-morbidity of hoarding and depression.

## Introduction

Hoarding disorder (HD) is a psychiatric condition characterized by indecision and difficulty and distress associated with discarding possessions, often accompanied by the excessive acquisition of items, that leads to the accumulation of high volumes of clutter (American Psychiatric Association, 2013). HD and compulsive hoarding behavior have a profound negative impact on functioning and health, and have been shown in several studies to affect quality of life (QoL; e.g., Saxena et al., 2011; Tolin et al., 2019; Nutley et al., 2021, 2022). Hoarding symptoms typically emerge during adolescence but often do not fully manifest until mid- to late-life, when the incidence of clinically significant hoarding symptoms rises from 2% to around 6% (Cath et al., 2017).

Although prior research has examined the relationship between hoarding and QoL as a general construct (e.g., Ong et al., 2015), our understanding of this relationship is currently incomplete as most studies published to date have focused on hoarding symptoms in the context of obsessive–compulsive disorder (OCD) rather than hoarding as an independent disorder. Further complicating the picture, these studies have used a variety of measures, and the specific aspects or domains of QoL examined also vary by study.

For example, of eight studies published between 2009 and 2022 examining the relationship between hoarding and QoL, only two were in participants with primary HD (Ong et al., 2016; Tolin et al., 2019), as hoarding was classified as a distinct diagnosis in the Diagnostic and Statistical Manual of Mental Disorders, 5th Edition (DSM-5) in 2013, while the other six were in primary OCD participants (Huppert et al., 2009; Albert et al., 2010; Fontenelle et al., 2010; Saxena et al., 2011; Subramaniam et al., 2014; Schwartzman et al., 2017). The studies also utilized a variety of QoL measures, including the Short-Form Health Survey-36 (SF-36), the Lehman Quality of Life Interview-Short Form, the EuroQol 5 Dimension Scale (EQ-5D), and the Quality of Life Enjoyment and Satisfaction Questionnaire (QLESQ).

The first of these studies (Huppert et al., 2009) examined hoarding behavior and QoL in a sample of individuals with OCD using the QLESQ, the SF-36, and the Social Adjustment Scale-Self Report (SAS-SR). The QLESQ is composed of a General Activities score of overall satisfaction, with seven sub-domain scores pertaining to Physical Health, Emotional Well-Being, Household Duties, Leisure Time Activities, Social Relations, Work, and School. The study found negative correlations between hoarding symptoms on the Obsessive–Compulsive Inventory-Revised (OCI-R) and the (1) QLESQ total, social, and family scales, as well as (2) impairment on the work (role functioning due to emotional problems) and social functioning scales of the SF-36, and (3) the total and social scores of the SAS-SR. However, the authors noted that depression accounted for much of the observed relationships between hoarding and QoL in their analyses.

A second study (Fontenelle et al., 2010) found that hoarding severity, as measured by the Saving Inventory-Revised (SI-R), in a model that also included depressive symptoms [measured *via* the Beck Depression Inventory (BDI)] along with employment status, predicted 62% of the variance in social functioning as assessed by the SF-36. The SF-36 consists of 8 domains divided into two major dimensions. The first dimension, physical health, includes the following domains: vitality, physical functioning, bodily pain, general health, and physical role, whereas the mental health dimension includes emotional role, social functioning, and mental health, with lower scores on each dimension indicating greater disability (Lins and Carvalho, 2016). Although hoarding symptom severity was independently associated with SF-36 scores, as with the Huppert et al. (2009) study, depression accounted for the majority of the variance in this model. The authors additionally speculated that possible lack of insight in those with hoarding symptoms could have impacted their ratings of QoL.

A third study published in 2010 by Albert et al. (2010) did not find significant correlations between any of the SF-36 subscales and the presence of hoarding obsessions and compulsions as measured by the Yale-Brown Obsessive-Compulsive Scale (Y-BOCS) Symptom Checklist. In a fourth study, Saxena et al. (2011), in OCD patients with or without co-morbid hoarding symptoms, examined the relationship between hoarding symptoms and QoL as measured by the Lehman Quality of Life Interview-Short Form [comprised of two domains: (1) objective, which includes Daily Activities, Family Contact, Social Contact, Financial Adequacy, Victimization, Arrests, and Amount Spent and (2) subjective, which includes Daily Activities, Family Contact, Social Relations, Finances, Safety, Life Satisfaction, Living Situation, and Health]. This study found that individuals with both OCD and hoarding had significantly lower scores on satisfaction with safety and living arrangements and significantly higher scores on victimization than did those with non-hoarding OCD, with the two groups not differing on depression or anxiety, suggesting that the findings of functional impairment were independent of affective severity.

Next, a study by Subramaniam et al. (2014) using the EuroQol 5 (which assesses Mobility, Self-Care, Usual Activities, Pain/Discomfort, and Anxiety/Depression) to measure health-related QoL found that patients who had both OCD and hoarding had lower QoL scores than did patients with only OCD or only hoarding, and were also more likely to report mobility and pain problems. Furthermore, the authors employed the Sheehan Disability Scale (SDS) as a measure of functioning, and found impairment in the home management and social life domains in patients with both OCD and hoarding compared to OCD without hoarding. Finally, Schwartzman et al. (2017) found that hoarding was the only subtype on the Y-BOCS to be significantly associated with household-related QoL on the QLESQ (but not social relations, leisure activities, or physical health) after controlling for OCD and depression severity.

In contrast, only two studies have examined the relationship of hoarding and QoL independent of underlying OCD. Ong et al. (2016), in a sample of 102 individuals with clinically significant hoarding symptoms determined *via* SI-R scores, found a negative correlation between hoarding severity and QoL on the QLESQ. However, after controlling for anxiety and depression, hoarding severity was no longer significantly associated with QoL. The authors also found that depressive symptoms on the BDI significantly predicted hoarding severity.

Finally, in the most recent study, Tolin et al. (2019) used the SF-36 to examine the relationship between hoarding severity and QoL in a group of 54 participants with primary HD and 24 healthy controls. The study found that after controlling for stress and affective symptoms, compared to healthy controls, those with HD had significantly decreased QoL in several domains, including role limitations due to emotional problems, emotional well-being, vitality, and general health. Across the entire sample, hoarding severity on the SI-R significantly improved regression models predicting impairment in an array of domains (social functioning, emotional well-being, role limitations due to physical health, role limitations due to emotional problems, vitality, and general health QoL), with depression, anxiety, and stress as co-variates. However, when the same analysis was performed within the HD sample alone, with co-morbid depression controlled for, only the model fit for vitality was significantly improved when hoarding severity was included.

Overall, the findings from these studies, which are summarized in Supplementary Table 1, suggest that hoarding symptoms may be negatively associated with multiple aspects of QoL. However, interpretation of the findings is somewhat limited by the fact that most studies were conducted in OCD samples, and thus could not distinguish the unique contributions of obsessive–compulsive symptoms from hoarding symptoms upon QoL. Additionally, depressive symptoms played a prominent role in impacting QoL in several of the studies, appearing to explain some, if not most, of the effects of hoarding symptoms. Accordingly, the relationship of hoarding and obsessive–compulsive symptoms with depressive symptoms, and their subsequent impact on QoL, should be explicitly examined.

Because most of the prior work on hoarding and QoL was in OCD patient populations, in this study we sought to examine and compare the relationships between QoL and hoarding and obsessive–compulsive symptomatology as separate constructs in a primarily non-clinical population (acquired online for a related study by our group) that we opportunistically analyzed. Despite some co-occurrence of these symptom profiles, it is now believed that HD is neurobiologically distinct from OCD, with only around 20% of patients with HD exhibiting co-morbid OCD (Frost et al., 2011). Prior studies in OCD patients have found impairments across multiple QoL domains (e.g., Norberg et al., 2008a; Subramaniam et al., 2014), including social functioning (Koran et al., 1996), role limitations due to emotional problems (Bobes et al., 2007), and activities of daily living (Lochner et al., 2003). Associations have also been identified between OCD symptom severity as measured by the Y-BOCS and the physical and mental components of the SF-36 (Albert et al., 2010), although few studies have directly compared obsessive–compulsive symptoms to primary hoarding symptoms independent of OCD. Moreover, as depression is highly co-morbid with both hoarding and OCD (e.g., Frost et al., 2011; Tibi et al., 2017), we also sought to explore how affective symptoms might contribute to any observed relationships between QoL and hoarding or obsessive–compulsive symptomatology. Multiple studies have established an association between impaired QoL and depressive symptoms (e.g., Norberg et al., 2008b; Sivertsen et al., 2015), but fewer studies have characterized how hoarding symptoms might interact with depression to impact QoL, and it is important to determine the extent to which affective symptoms might drive or explain the previously identified relationships between hoarding and decreased QoL.

Although the SF-36, which was used in several of the prior studies examining the effect of hoarding symptoms on QoL, is a validated and commonly used self-report survey, it does not fully encompass certain aspects of QoL that may be relevant to hoarding symptomatology. The SF-36 captures many aspects of health-related QoL, especially physical health dimensions, but relationships with family, success in work or academics, and comfort and livability of the home environment, among others, are not captured by this instrument, and these domains may be especially relevant to hoarding and/or obsessive–compulsive symptomatology. As a result, despite the previous work that has been conducted examining QoL, the relationship between hoarding and these specific areas of QoL remains inconclusive (Ong et al., 2015).

Given the scarcity of literature on the relationships between primary hoarding symptoms, OCD symptoms, and specific QoL domains, the objective of this study was to further explore these relationships, with particular attention to how hoarding symptoms distinctly affect QoL compared to obsessive–compulsive symptoms. We also examined the potential confounding of any observed relationships between hoarding and QoL by co-occurring affective symptoms. We chose to assess QoL using the Quality of Life Inventory (QoLI; Frisch et al., 1992), a psychometrically validated measure of overall physical and emotional well-being that assesses the “Sweet 16” areas of life, including areas such as work, love, and goals and values in addition to physical and emotional health (Frisch et al., 1992). The QoLI examines both an individual’s satisfaction in 16 areas of life as well as how importantly they rate each one, which allows for an overall weighted score and a factored score for each area and leads to a more detailed and comprehensive view of QoL.

We hypothesized that the presence of more severe hoarding symptomatology would be associated with reduced QoL, and that compared to patterns seen with obsessive–compulsive symptomatology, hoarding symptomatology would be associated with unique aspects of QoL. Specifically, we hypothesized that hoarding symptomatology would be most strongly associated with decreased QoL in the home environment and family domains, whereas obsessive–compulsive symptomatology would be more strongly associated with other QoL domains. We also hypothesized, consistent with previous studies, that depression symptomatology would explain much, if not all, of the relationship between hoarding symptom severity and QoL.

## Materials and Methods

### Participants

Participants were recruited from Amazon Mechanical Turk (mTurk), an online crowdsourcing platform accessible to the general public. The study was reviewed and approved by the University of Florida’s Institutional Review Board (IRB) and informed consent was obtained from all participants. Participants were eligible for inclusion if they were adult residents of the United States, 18 years of age or older. Participants saw the following description when viewing the study on Amazon mTurk: “Complete questionnaires on how you think and feel – takes about 30 minutes.” The study was titled “Cognitive Insight Questionnaire” and a total of 90 minutes was allotted for participants to complete the survey. The project was originally intended to gather data on relationships between cognitive insight and metacognition along with pertinent psychiatric symptomatology and QoL. We excluded individuals who did not correctly answer two check questions, answered fewer than 99% of the 156 total questions, or who completed the entire survey in less than 4 minutes, indicating automatic rather than thoughtful responding. Seven individuals indicated non-binary gender, and were excluded for analysis purposes due to the small sample size in this group. After removing those who did not meet our inclusion criteria, we obtained a final total sample of 2100.

### Assessments

The survey was created with Qualtrics software and published on mTurk for 4 days. The survey assessed basic demographic information including age, sex, race, education, marital status, employment status, and income, and included the following validated self-report questionnaires: the Hoarding Rating Scale, Self-Report (HRS-SR), OCI-R, Generalized Anxiety Disorder 7-Item Scale (GAD-7), Patient Health Questionnaire-9 (PHQ-9), and QoLI. We used recommended and established guidelines in the literature to score all scales, and to determine severity and clinical cut-off levels for the psychiatric scales.

The HRS-SR consists of 5 questions that assess clutter, difficulty discarding, acquisition, distress, and interference related to hoarding, each rated on a scale of 0–8 (0 = not problematic, 2 = mild, 4 = moderate, 6 = severe, 8 = extreme), for a minimum of 0 and maximum of 40. We considered a total score of 14 or higher to be suggestive of probable hoarding, based on prior cutoffs in the literature (Tolin et al., 2010).

The OCI-R is an 18-item instrument, with each item measured on a Likert scale (0 = not at all, 1 = a little, 2 = moderately, 3 = a lot, 4 = extremely), for a minimum of 0 and maximum of 72. The OCI-R is comprised of six domains, with three questions contributing to each domain: washing, checking, ordering, obsessing, hoarding, and neutralizing. We removed the hoarding-related questions to reduce overlap with the HRS-SR and potential inflation of scores being driven by the hoarding dimension, for a final total of 15 items (minimum score of 0, maximum of 60). We considered a total score of 21 or higher to indicate the likely presence of OCD, per previously established guidelines (Foa et al., 2002). We retained a strict threshold of 21 after item adjustment in order to avoid potentially overestimating OCD prevalence.

The GAD-7 is a screening tool for generalized anxiety disorder (GAD), aimed at assessing the presence of 7 criteria for GAD over the previous 2 weeks. Each item is scored on a scale of 0–3 (0 = not at all, 1 = several days, 2 = more than half the days, 3 = nearly every day) and individual item scores are summed to create a total score, for a minimum of 0 and maximum of 21, with higher scores indicating more severe symptoms. We used a total score of 10 or above to suggest a clinically significant level of anxiety symptoms (Spitzer et al., 2006).

The PHQ-9 is a 9-item measure intended to be a brief screen for depression and is scored similarly to the GAD-7, with individual items on a scale of 0–3 (0 = not at all, 1 = several days, 2 = more than half the days, 3 = nearly every day) for a minimum of 0 and maximum of 27 and higher scores indicating more severe symptoms. We used a total score of 10 or above as suggesting a clinically significant level of depressive symptoms (Kroenke et al., 2001).

The QoLI measures 16 areas of life and incorporates both importance of (0 = not important, 1 = important, 2 = extremely important) and satisfaction with each area (−3 = very dissatisfied, −2 = somewhat dissatisfied, −1 = a little dissatisfied, +1 = a little satisfied, +2 = somewhat satisfied, +3 = very satisfied). The 16 areas include health, self-esteem, goals and values, money, work, play, learning, creativity, helping, love, friends, children, relatives, home, neighborhood, and community. The importance and satisfaction scores are multiplied by one another to generate a weighted satisfaction rating, with scores ranging from −6 to 6 [classified as very low QoL = −6–0.8, low = 0.9–1.5, average = 1.6–3.5, and high = 3.6–6 based on *t*-score distribution and percentile scores from a non-clinical standardization sample (Frisch, 1992; Grant et al., 1994)]. We totaled the weighted satisfaction rating for each area and divided by the number of areas (with non-zero scores) to compute an overall QoLI raw score (higher indicating better QoL) for each respondent (Frisch et al., 1992). Areas of life that were rated as not important were not incorporated into the overall QoLI raw score.

### Analyses

First, demographic characteristics of the sample were summarized using frequencies and distributions, and individual items for each of the psychiatric questionnaires were summed to generate total quantitative scores. All demographic variables were converted from multi-categorical to dichotomous for inclusion in linear regression models. We first assessed the correlations between the demographic variables, psychiatric symptom scale total scores, and total QoL scores to evaluate which variables to include as covariates. This yielded age, income, employment status, education, and marital status, as well as race and sex due to their relationship with our psychiatric predictor variables, as covariates in the regression models. We also created dichotomous/binary variables to indicate the presence or absence of clinically significant symptoms for each respondent based on established cutoff scores for each diagnosis (specified above).

A factor analysis with oblique promax rotation using the weighted satisfaction scores (importance × satisfaction) obtained from the 16 items of the QoLI was conducted using the entire sample to identify relevant QoL groupings for use in subsequent analyses. Items were considered to load on a given factor if they had a factor loading of >0.40. An eigenvalue >1 was used to determine the relevant number of factors. If an item cross-loaded onto more than 1 factor, it was assigned to the factor with the strongest loading unless any of the loadings were within 0.05 of one another, in which case it was assigned to both factors. After identifying the appropriate factor structure, the items within each factor/domain were examined, and each factor was named accordingly based on the components. Next, for each participant, a weighted sum score for each QoL factor/domain was computed. The weighted sum scores were calculated as the sum of the weighted satisfaction scores of the component QoL subdomains for each of the derived factors, averaged by the number of component subdomains within the QoL factor.

The relationships between the outcomes of interest (e.g., the weighted sum scores for each factor-derived QoL category) and predictors of interest (e.g., hoarding, obsessive–compulsive, depression, and anxiety symptoms) were first assessed using bivariate Pearson correlations to determine the strength of associations between variables and whether multicollinearity was potentially present. We employed a cutoff of *r* ≥ 0.70 as indicative of multicollinearity between variables, and excluded the variable less related to QoL (based on the magnitudes of the overall bivariate Pearson correlations with the QoL domains).

We next characterized the sample by performing an independent samples *t*-test to compare those who had clinically significant hoarding symptoms to those without, using our dichotomous variables based on clinical cutoff scores. One-way ANOVAs were also performed to compare hoarding only, obsessive–compulsive only, and hoarding + obsessive–compulsive symptom groups on QoL as well as hoarding only, depression only, and hoarding + depression symptom groups (Supplementary Tables 5–7).

Several sequential multiple linear regression models were then conducted with the QoL domains identified through factor analysis as outcomes. First, the HRS-SR and OCI-R were included as predictors in separate models to determine their individual contributions to QoL, with the demographic variables included as covariates (Table 2). Next, the HRS-SR and OCI-R were included in the same model to control for one another’s effects on QoL, with the demographic variables as covariates (Table 3). Lastly, we added the PHQ-9 to the prior model with both the HRS-SR and OCI-R and the covariates to determine the impact of depression symptomatology on QoL (Table 4). We deemed a *p*-value of < 0.05 as statistically significant. Mediation analyses were then performed using the Process v3.4 macro (Hayes, 2013) in IBM SPSS Statistics 26 (IBM Corp, 2019) with 5000 bootstrapped samples and 95% confidence intervals to determine the PHQ-9’s role as a potential partial or complete mediator of the effects of the OCI-R and HRS-SR on QoL as well as the magnitudes of the relationships between the OCI-R, HRS-SR, and PHQ-9 and the direct and indirect effects of the OCI-R and HRS-SR on QoL. Factor analysis was performed with Stata/SE 16.1 software (StataCorp, 2019), and demographics, correlations, regressions, and graphs with IBM SPSS Statistics 26.

**TABLE 1:** Demographics of the sample (*n* = 2100).

	Whole sample (*n* = 2100)	HD only (*n* = 100)	OCD only (*n* = 76)	MDD only (*n* = 98)	GAD only (*n* = 64)	2+ disorders (*n* = 492)	No psychiatric symptomatology (*n* = 1270)
**Age**							
18–44 years	74.3% (*n* = 1561)	73.0% (*n* = 73)	77.6% (*n* = 59)	74.5% (*n* = 73)	85.9% (*n* = 55)	86.8% (*n* = 427)	68.8% (*n* = 874)
45+ years	25.7% (*n* = 539)	27.0% (*n* = 27)	22.4% (*n* = 17)	25.5% (*n* = 25)	14.1% (*n* = 9)	13.2% (*n* = 65)	31.2% (*n* = 396)
**Sex**							
Female	59.7% (*n* = 1254)	68.0% (*n* = 68)	63.2% (*n* = 48)	59.2% (*n* = 58)	64.1% (*n* = 41)	60.2% (*n* = 296)	58.5% (*n* = 743)
**Race**							
White	77.5% (*n* = 1627)	74.0% (*n* = 74)	76.3% (*n* = 58)	73.5% (*n* = 72)	76.6% (*n* = 49)	70.3% (*n* = 346)	80.9% (*n* = 1028)
**Education**							
College degree	65.6% (*n* = 1377)	61.0% (*n* = 61)	64.5% (*n* = 49)	57.1% (*n* = 56)	67.2% (*n* = 43)	54.5% (*n* = 268)	70.9% (*n* = 900)
**Marital status**							
Married	53.0% (*n* = 1113)	57.0% (*n* = 57)	59.2% (*n* = 45)	62.2% (*n* = 61)	60.9% (*n* = 39)	47.4% (*n* = 233)	55.3% (*n* = 702)
**Income**							
< $75,000	74.2% (*n* = 1558)	78.0% (*n* = 78)	65.8% (*n* = 50)	86.7% (*n* = 85)	81.3% (*n* = 52)	81.3% (*n* = 400)	70.3% (*n* = 893)
≥ $75,000	25.8% (*n* = 542)	22.0% (*n* = 22)	34.2% (*n* = 26)	13.3% (*n* = 13)	18.8% (*n* = 12)	18.7% (*n* = 92)	29.7% (*n* = 377)
**Employment**							
Employed	79.7% (*n* = 1674)	83.0% (*n* = 83)	76.3% (*n* = 58)	69.4% (*n* = 68)	79.7% (*n* = 51)	77.2% (*n* = 380)	81.4% (*n* = 1034)
**Psychiatric assessments**							
PHQ-9	Mea*n* = 5.92, SD = 5.99, range = 0-27	Mea*n* = 4.16, SD = 2.82, range = 0-9	Mea*n* = 3.86, SD = 2.68, range = 0-9	Mea*n* = 13.03, SD = 3.05, range = 10-21	Mea*n* = 6.42, SD = 1.96, range = 1-9	Mea*n* = 13.53, SD = 5.83, range = 0-27	Mea*n* = 2.67, SD = 2.60, range = 0-9
GAD-7	Mea*n* = 5.12, SD = 5.31, range = 0-21	Mea*n* = 3.45, SD = 2.64, range = 0-9	Mea*n* = 4.22, SD = 2.65, range = 0-9	Mea*n* = 6.00, SD = 2.39, range = 0-9	Mea*n* = 12.02, SD = 2.29, range = 10-21	Mea*n* = 12.03, SD = 4.97, range = 0-21	Mea*n* = 2.21, SD = 2.41, range = 0-9
HRS-SR	Mea*n* = 6.93, SD = 7.97, range = 0-40	Mea*n* = 17.69, SD = 3.28, range = 14-28	Mea*n* = 4.88, SD = 4.39, range = 0-13	Mea*n* = 5.47, SD = 4.34, range = 0-13	Mea*n* = 4.39, SD = 4.07, range = 0-13	Mea*n* = 15.34, SD = 9.69, range = 0-40	Mea*n* = 3.19, SD = 3.63, range = 0-13
OCI-R	Mea*n* = 11.21, SD = 10.70, range = 0-57	Mea*n* = 10.24, SD = 5.35, range = 0-20	Mea*n* = 26.53, SD = 5.83, range = 21-47	Mea*n* = 9.47, SD = 5.09, range = 0-20	Mea*n* = 11.11, SD = 4.76, range = 1-20	Mea*n* = 23.32, SD = 12.18, range = 0-57	Mea*n* = 5.82, SD = 4.95, range = 0-20
QoLI (total QoL)	Mea*n* = 1.58, SD = 1.88, range = − 4.5–6.0	Mea*n* = 1.39, SD = 1.42, range = − 3.29–5.0	Mea*n* = 2.10, SD = 1.47, range = − 1.37–4.80	Mea*n* = − 0.34, SD = 1.58, range = − 4.0 to4.0	Mea*n* = 0.69, SD = 1.63, range = − 3.21–5.40	Mea*n* = 0.42, SD = 1.93, range = − 4.5–6.0	Mea*n* = 2.20, SD = 1.60, range = − 4.07–6.0
**Quality of Life Inventory (QoLI) total rating**							
Very Low (− 6.0–0.8)	33.7% (*n* = 707)	42.0% (*n* = 42)	21.1% (*n* = 16)	76.5% (*n* = 75)	50.0% (*n* = 32)	59.3% (*n* = 292)	19.7% (*n* = 250)
Low (0.9–1.5)	10.5% (*n* = 221)	11.0% (*n* = 11)	6.6% (*n* = 5)	11.2% (*n* = 11)	17.2% (*n* = 11)	12.4% (*n* = 61)	9.6% (*n* = 122)
Average (1.6–3.5)	42.4% (*n* = 891)	43.0% (*n* = 43)	57.9% (*n* = 44)	11.2% (*n* = 11)	29.7% (*n* = 19)	22.4% (*n* = 110)	52.3% (*n* = 664)
High (3.6–6.0)	13.4% (*n* = 281)	4.0% (*n* = 4)	14.5% (*n* = 11)	1.0% (*n* = 1)	3.1% (*n* = 2)	5.9% (*n* = 29)	18.4% (*n* = 234)
**Clinical symptomatology**							
% who met clinical cutoff for depression (PHQ-9 scores ≥ 10)	23.6% (*n* = 495)						
% who met clinical cutoff for anxiety (GAD-7 scores ≥ 10)	20.4% (*n* = 429)						
% who met clinical cutoff for hoarding (HRS-SR scores ≥ 14)	18.7% (*n* = 392)						
% who met clinical cutoff for OCD (OCI-R scores ≥ 21)	17.6% (*n* = 370)						
% who met clinical cutoffs for 1+ disorder	39.6% (*n* = 830)						
% who met clinical cutoffs for 2+ disorders	23.5% (*n* = 492)						

**TABLE 2:** Multiple linear regression comparing the HRS-SR and OCI-R as predictors of QoL in separate models (covariates not shown).

QoL domain	OCI-R beta coefficient (standardized, unstandardized)	*t*	*p*	Adjusted *R*-square, *F*-value	HRS-SR beta coefficient (standardized, unstandardized)	*t*	*p*	Adjusted *R*-square, *F*-value
Total	−0.154 (−0.027)	−7.30	<0.001	0.114, 34.923	−0.169 (−0.040)	−8.13	<0.001	0.120, 36.692
Success	−0.124 (−0.026)	−5.88	<0.001	0.119, 36.475	−0.145 (−0.042)	−7.00	<0.001	0.125, 38.496
Enrichment	−0.136 (−0.024)	−6.18	<0.001	0.042, 12.376	−0.127 (−0.030)	−5.87	<0.001	0.040, 11.893
Environment	−0.104 (−0.021)	−4.78	<0.001	0.063, 18.633	−0.125 (−0.033)	−5.81	<0.001	0.068, 20.079
Family	−0.093 (−0.020)	−4.54	<0.001	0.165, 52.796	−0.112 (−0.032)	−5.54	<0.001	0.169, 54.297

*Unstandardized beta coefficients are given in parentheses.*

**TABLE 3:** Multiple linear regression including both the HRS-SR and OCI-R in the same model as predictors of QoL (covariates not shown).

QoL domain	OCI-R beta coefficient (standardized, unstandardized)	*t*	*p*	HRS-SR beta coefficient (standardized, unstandardized)	*t*	*p*	Adjusted *R*-square, *F*-value
Total	−0.082 (−0.014)	−3.16	0.002	−0.122 (−0.029)	−4.75	<0.001	0.124, 33.866
Success	−0.057 (−0.012)	−2.22	0.03	−0.112 (−0.032)	−4.38	<0.001	0.127, 34.832
Enrichment	−0.092 (−0.016)	−3.38	0.001	−0.074 (−0.018)	−2.78	0.005	0.045, 11.896
Environment	−0.046 (−0.009)	−1.72	0.09	−0.098 (−0.026)	−3.71	<0.001	0.069, 18.191
Family	−0.041 (−0.009)	−1.61	0.11	−0.089 (−0.025)	−3.55	<0.001	0.169, 48.589

*Unstandardized beta coefficients are given in parentheses.*

**TABLE 4:** Multiple linear regression including both the HRS-SR and OCI-R in the same model, with the addition of the PHQ-9, as predictors of QoL (covariates not shown).

QoL domain	OCI-R beta coefficient (standardized, unstandardized)	*t*	*p*	HRS-SR beta coefficient (standardized, unstandardized)	*t*	*p*	PHQ-9 beta coefficient (standardized, unstandardized)	*t*	*p*	Adjusted *R*-square, *F*-value
Total	0.136 (0.024)	5.90	<0.001	0.030 (0.007)	1.33	0.18	−0.619 (−0.194)	−29.03	<0.001	0.375, 126.996
Success	0.167 (0.036)	7.33	<0.001	0.044 (0.013)	1.99	0.05	−0.637 (−0.243)	−30.30	<0.001	0.393, 136.925
Enrichment	0.090 (0.016)	3.48	0.001	0.051 (0.012)	2.06	0.04	−0.515 (−0.163)	−21.58	<0.001	0.218, 59.643
Environment	0.074 (0.015)	2.74	0.006	−0.015 (−0.004)	−0.57	0.57	−0.340 (−0.120)	−13.63	<0.001	0.144, 36.382
Family	0.094 (0.020)	3.73	<0.001	0.004 (0.001)	0.19	0.85	−0.380 (−0.146)	−16.44	<0.001	0.264, 76.380

*Unstandardized beta coefficients are given in parentheses.*

## Results

### Characteristics of the Sample

The final sample consisted of 2100 adult participants, with approximately three-fourths in the young to middle age groups (ages 18–44). Nearly 60% were female and over three-quarters were white. The sample was fairly well-educated, with roughly two-thirds of participants having attended college. Of note, a much higher percentage endorsed clinically significant levels of hoarding and obsessive–compulsive symptoms on the HRS-SR (18.7%) and OCI-R (17.6%), respectively, than anticipated based on population prevalences (Arditte et al., 2016; Cath et al., 2017). Nearly 40% of the sample met cutoff criteria for at least 1 psychiatric disorder (HD, OCD, depression, or anxiety), and nearly half the sample had low or very low QoL. Comparing by group, nearly three-quarters of those who met the criteria for only MDD and none of the other psychiatric disorders endorsed very low QoL, which was a higher proportion than the other groups. Overall QoL (−0.34) was also lower in the MDD-only group compared to the others, indicating that depressive symptoms were a primary driver of reduced QoL in this sample. Additionally, there were lower rates of being employed and having a college degree and lower average income in the MDD-only group. Complete characteristics are depicted in Table 1.

### Factor Analysis

Factor analysis of the 16 QoLI domains yielded a scree plot and eigenvalues suggesting a four-factor model, with groupings that we labeled ***success*** (comprised of health, self-esteem, goals and values, money, and work), ***enrichment*** (composed of play, learning, creativity, helping, and friends), ***environment*** (consisting of home, neighborhood, and community), and ***family*** (encompassing love, children, and relatives). Factor loadings are indicated in Supplementary Table 2. Each domain loaded cleanly onto a single factor, with no cross-loadings. Across the entire sample, we found the success QoL scores to be the lowest (mean = 0.94, SD = 2.29), followed by the environment (mean = 1.49, SD = 2.11) and enrichment (mean = 1.58, SD = 1.90) scores, with scores ranging from −6 to 6. Family QoL was the highest rated of the four QoL domains in the overall sample (mean = 2.16, SD = 2.29). Males and females in the overall sample significantly differed in the environment and family QoL domains, whereby females exhibited higher QoL than males in these categories (*p* < 0.05). However, males and females did not significantly differ in regards to total, success, or enrichment QoL (Supplementary Table 3).

### Relationships Between Quality of Life and Psychiatric Symptom Severity

Bivariate Pearson correlations showed that all correlations between psychiatric symptom subscales and each QoL domain were statistically significant (*p* < 0.01; Supplementary Table 4). All of these correlations were negative, indicating that the higher the score on each of the psychiatric symptom measures, the lower the QoL. The strongest correlations were between the QoL domains and the PHQ-9 and GAD-7 subscales, relative to the HRS-SR and OCI-R scales. In general, the success and enrichment QoL domains were more strongly correlated with the psychiatric symptom subscales than were the environment and family QoL domains. We also found that the GAD-7 and PHQ-9 were highly correlated with one another (>0.8), and therefore only the PHQ-9 was included in subsequent analyses due to its stronger relationships with QoL metrics than the GAD-7, and to avoid multicollinearity.

### Quality of Life by Subgroup

Characterizing QoL and psychiatric symptomatology in hoarding and non-hoarding groups (based on HRS-SR scores) revealed that participants who endorsed a clinically significant level of hoarding on the HRS-SR (i.e., a score ≥14) had significantly decreased QoL across all domains and significantly increased psychiatric symptomatology compared to subjects without clinically significant hoarding (Supplementary Table 5). However, comparing hoarding-only to depression-only, those who endorsed a clinical level of depression symptomatology had significantly lower QoL across all domains than those with solely hoarding symptomatology, while individuals with clinical levels of both hoarding and depression were in the middle relative to hoarding or depression only (Supplementary Table 6). Comparing hoarding-only to obsessive–compulsive-only, hoarding-only generally had lower QoL across domains than obsessive–compulsive-only, with the hoarding + obsessive–compulsive group being about the same or higher in QoL than the obsessive–compulsive-only group (Supplementary Table 7).

### Linear Regression Models

Multiple linear regression models including age, sex, race, income, employment status, education, and marital status as covariates, with either quantitative HRS-SR or OCI-R scores as predictors of QoL domains, revealed that hoarding and obsessive–compulsive symptomatology were individually negatively associated with all aspects of QoL, with hoarding symptom severity more strongly associated with lower scores in most QoL domains than OCD symptom severity (Table 2; beta values, *t*-statistics, and *p*-values for covariates not shown for brevity). Each overall model (including covariates) was statistically significant (*p* < 0.05), with adjusted *R*-squared and *F*-values highest for the models predicting total, success, and family QoL compared to enrichment and environment QoL.

When the HRS-SR and OCI-R were included together as predictors in the same model, along with the covariates specified above, hoarding symptom severity was significantly associated (*p* < 0.05) with all QoL domains, while obsessive–compulsive symptom severity was significantly associated only with total QoL (*p* = 0.002), success QoL (*p* = 0.03) and enrichment QoL (*p* = 0.001) (Table 3). Each overall model (including covariates) was statistically significant (*p* < 0.05), with adjusted *R*-squared and *F*-values again highest for the models predicting total, success, and family QoL compared to enrichment and environment QoL.

Multiple linear regression models that included the HRS-SR, OCI-R, and the PHQ-9 jointly as predictors of QoL revealed that hoarding symptoms were no longer significantly associated (*p* > 0.05) with total, environment, or family QoL, and only marginally associated with success and enrichment, while obsessive–compulsive symptoms were now significantly correlated with all QoL domains (Table 4). Each overall model (including covariates) was statistically significant (*p* < 0.05). The PHQ-9 exerted the strongest effect on all QoL domains compared to the HRS-SR or OCI-R, and the model fit when depressive symptoms were included greatly improved, as indicated by the adjusted *R*-square values.

Sequential linear regression models were also conducted with the subcomponents of environment QoL (home, neighborhood, community) and family QoL (love, children, relatives) as secondary *post hoc* analyses in order to assess individual aspects specifically related to social and home functioning. We found that both the OCI-R and HRS-SR were individually significantly associated with lower QoL on all subcomponents, with the exception of the OCI-R and children. Overall, based on the beta coefficients and *t*-statistics, the HRS-SR exerted a stronger impact than the OCI-R on all of the subcomponents except community and displayed a particularly strong relationship with home (Supplementary Table 9). Including the OCI-R and HRS-SR in the same model indicated that the HRS-SR displayed greater associations with the QoL subcomponents (with the exception of community) compared to the OCI-R and especially with home (Supplementary Table 10). However, as was seen in our previous analyses, incorporating depression into the model weakened the effects of the HRS-SR on all of the QoL subcomponents (Supplementary Table 11), although it was still significantly negatively related to home. The PHQ-9 again explained the majority of the variance compared to the HRS-SR and OCI-R.

### Interaction Between Hoarding and Depression Severity

**FIGURE 1:**
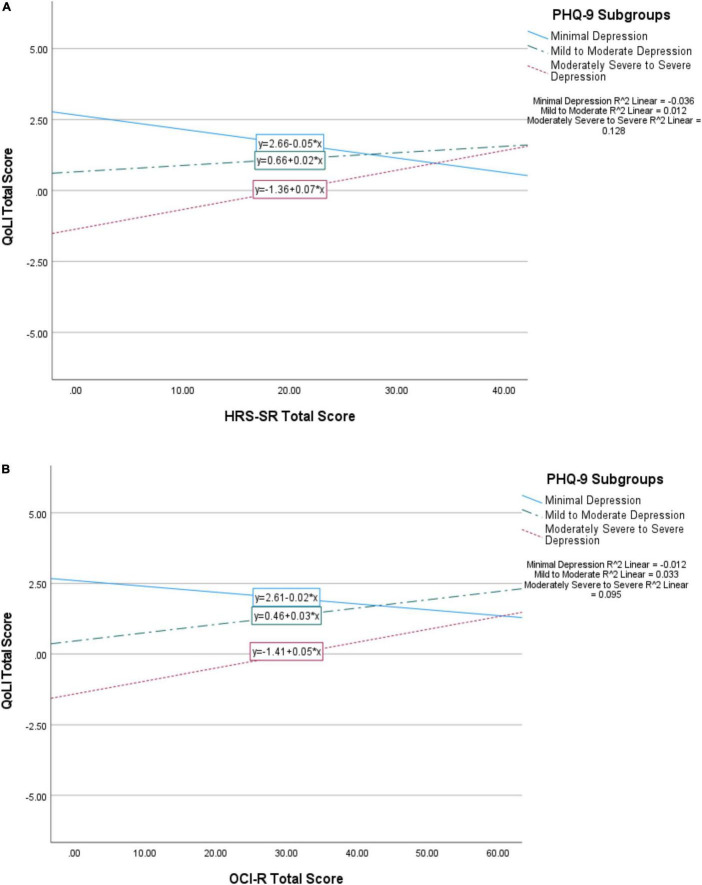
Interaction of PHQ-9 subgroups (minimal depression, mild to moderate depression, moderately severe to severe depression) with HRS-SR **(A)** and OCI-R **(B)** scores upon total QoL. Minimal depression is represented by a solid line, mild to moderate by a dotted and dashed line, and moderately severe to severe by a dashed line. The symbol * represents multiplication.

Because the relationships between the OCI-R and HRS-SR and the QoL domains were in a positive direction (more hoarding and/or OCD symptom severity was associated with better QoL) with the addition of the PHQ-9 to the regression models, we further probed the nature of the interaction between depression, hoarding, and OCD symptom severity. We divided PHQ-9 scores into three groups based on established cutoffs [Kroenke et al., 2001; 0–4 = minimal depression (*n* = 1110 or 52.9%); 5–14 = mild to moderate depression (*n* = 768 or 36.6%); 15+ = moderately severe to severe depression (*n* = 222 or 10.6%)] and graphed the interaction of these subgroups with OCI-R and HRS-SR scores. We found that those with moderate or severe depressive symptoms had better QoL with the presence of more hoarding symptoms, while those with minimal depressive symptoms had worsened QoL with higher hoarding scores (Figure 1A). Similar relationships were seen between depression and OCD symptom severity (Figure 1B). To further explore these relationships, as a *post hoc* analysis we next split the sample into depressed and non-depressed groups based on the previously specified PHQ-9 cutoff of 10 and re-ran the regression analyses with HRS-SR predicting QoL domains. These results were similar to those seen in Figure 1, whereby hoarding symptoms displayed positive relationships with all QoL domains in the depressed group, but had negative relationships in the non-depressed group (Supplementary Table 8; covariates not shown).

### Mediation Analyses

**FIGURE 2:**
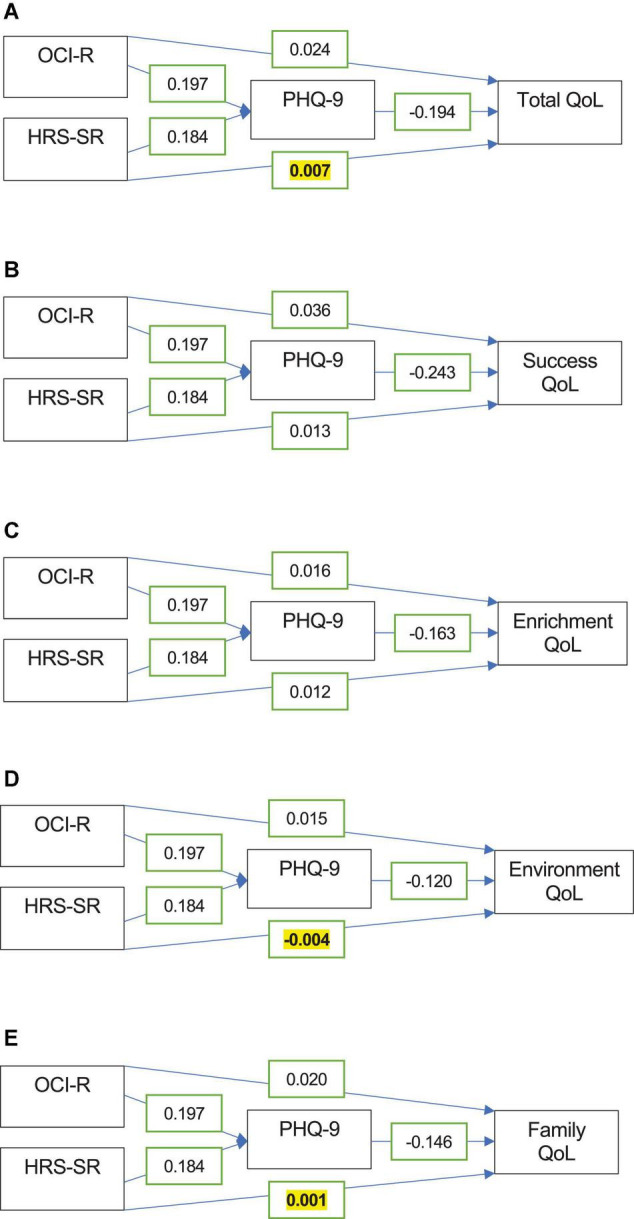
Mediation analysis models with the PHQ-9 as a mediator of the OCI-R and HRS-SR’s relationships with total **(A)**, success **(B)**, enrichment **(C)**, environment **(D)**, and family QoL **(E)**. Coefficients for the relationships between variables are displayed in the boxes. All bivariate relationships were statistically significant, except for the direct effect of the HRS-SR on total, environment, and family QoL (*p* > 0.05, bolded and highlighted in yellow).

Multiple mediation models including the PHQ-9 as a proposed mediator of the effects of the OCI-R and HRS-SR on QoL were then conducted as a follow-up to the regression analyses (Figure 2). These results confirmed those of the regression analyses, where the direct effect of the HRS-SR on total, environment, and family QoL were no longer significant (*p* > 0.05), indicating that the PHQ-9 acted as a complete mediator in these models. The PHQ-9 also explained much of the impact of the HRS-SR on success and enrichment QoL, acting as a partial mediator in these models (Figure 2).

## Discussion

The results of this study indicate that, in a large sample of 2100 adults, hoarding and obsessive–compulsive symptomatology were each individually negatively associated with all aspects of QoL (total, success, enrichment, environment, and family) as measured by the QoLI, with hoarding generally exerting a stronger impact than obsessive–compulsive symptoms. We also found that, as hypothesized, hoarding and obsessive–compulsive symptomatology were associated with different aspects of QoL after accounting for the presence of the other. Hoarding symptoms, but not obsessive–compulsive symptoms, were associated with reduced QoL in all four domains. In contrast, obsessive–compulsive symptomatology was associated with reduced QoL in the domains of success (health, self-esteem, goals and values, money, and work) and enrichment (play, learning, creativity, helping, and friends), but not significantly associated with environment (home, neighborhood, and community) and family (love, children, and relatives) after controlling for covariates.

When adding depressive symptoms into the model, we found that the relationships between hoarding symptoms, obsessive–compulsive symptoms, and QoL became more complex. As expected, depression symptomatology had the strongest associations with QoL in all domains and accounted for the majority of reduced QoL. Because the PHQ-9 was highly correlated with the GAD-7, we decided to use solely the PHQ-9 to avoid multicollinear effects and because of its stronger overall correlations with QoL and the higher co-morbidity of depression with hoarding. Unexpectedly, although only statistically significant for obsessive–compulsive symptoms, both hoarding and obsessive–compulsive symptoms showed positive associations with the QoL domains when depression was included in the model. Examining these relationships by depression subgroup indicated that, in fact, hoarding and obsessive–compulsive symptoms were positively correlated with QoL in the context of clinically meaningful depressive symptoms, while they had the hypothesized negative association with QoL only in the context of minimal depressive symptoms. These findings, while unexpected, suggest that in this sample, both hoarding and obsessive–compulsive symptoms potentially acted as a buffer in the context of more severe depressive symptoms. While we do not have a way to test this hypothesis in the current sample, these findings are worth replicating and investigating further in separate samples.

Our results highlight the importance of considering specific features of QoL in assessing the impact of hoarding and obsessive–compulsive symptoms and provide evidence to support the hypothesis that hoarding symptomatology has a unique, and in some cases, stronger impact on QoL when compared to obsessive–compulsive symptomatology. As predicted, hoarding symptomatology was more strongly associated with the environment and family domains than was obsessive–compulsive symptomatology, possibly due to the excessive acquisition of items and accumulation of clutter that is common among individuals with HD. These symptoms not only prevent the use of living spaces and can become hazardous, unsanitary, and functionally impairing, but could also extend to individuals being reluctant to invite friends or family to their home due to their living situation and the accompanying shame or embarrassment (International OCD Foundation, 2014). In turn, patients may feel socially isolated, and the hoarding behavior could persist or go undiagnosed, thereby precluding proper intervention. Hoarding symptomatology is known to strain the relationships that individuals share with their significant others and loved ones (e.g., leading to conflict or frustration) as well as negatively impact the lives of others in the household (Tolin et al., 2008). It is also known that hoarding behavior is associated with lower rates of marriage and higher rates of divorce (Steketee and Frost, 2003; Archer et al., 2019) as well as a tendency to live alone, which could further negatively affect family QoL.

In contrast, compared to hoarding symptomatology, obsessive–compulsive symptomatology in this sample was more strongly associated with decreases in enrichment QoL than with environment and family QoL. This may be because the presence of time-consuming and distressing obsessions and/or compulsions could interfere especially with one’s ability to maintain friendships or achieve their maximum potential with learning or creativity. On the other hand, these symptoms, compared to hoarding, may possibly be slightly less impairing in the home or family situation where symptoms are either accommodated, or alternatively, where parents or other loved ones familiar with their symptoms might be able to assist in the management of them in a more controlled setting.

As expected, depression symptomatology was most strongly correlated with all domains of QoL compared to other psychiatric symptoms. These findings are in line with those of Fontenelle et al. (2010), who found that depressive symptoms were the strongest predictor of social functioning, although hoarding symptoms also played a role. Other studies have also found depression symptoms to have a larger impact on QoL than other psychiatric symptoms (e.g., Subramaniam et al., 2014; Sagayadevan et al., 2018). However, our findings contrast with those of Tolin et al. (2019), who found effects of hoarding severity on multiple aspects of QoL (e.g., social functioning, emotional well-being, and vitality) that were independent of depression and anxiety, although physical health-related QoL on the SF-36 was mostly driven by affective symptoms. These differences might be partly attributed to the differences in QoL metrics and in the study samples – whereas Tolin et al. (2019) used a primarily treatment-seeking sample, presumably with insight into the impact of their hoarding symptoms, ours was a primarily non-clinical population, though with a bias toward individuals endorsing hoarding and obsessive–compulsive symptomatology. It would therefore be interesting to examine the role of insight in QoL and investigate its relationship to hoarding and affective symptomatology, particularly in clinical populations with hoarding.

Our findings have potential relevance to individuals who experience obsessive–compulsive or hoarding symptomatology. As depression is highly co-morbid with both HD and OCD, actively addressing depressive symptoms should be a priority for providers, as it may ultimately impact QoL in hoarding and obsessive–compulsive populations. Similarly, gaining a better understanding of how QoL is impacted by hoarding and obsessive–compulsive symptomatology and how depression symptomatology interacts with them may also lead to more informed and effective interventions to ultimately improve QoL in patients with these disorders and allow for more targeted interventions. In our sample, we found that QoL increased as hoarding severity increased in participants with mild, moderate, moderately severe, or severe depression symptomatology, as opposed to the pattern seen in those with minimal depression symptoms. This effect suggests that in the sample, hoarding symptoms could have acted as a compensatory or coping mechanism in the context of substantial depressive symptoms; for example, the possession and accumulation of items could provide a sense of safety and comfort, increasing QoL and partly mitigating the effects of depression. However, this hypothesis remains untested, and warrants further exploration.

### Limitations

Although our study provides information about the relationships between QoL and hoarding, obsessive–compulsive, and depressive symptoms, there are also some limitations inherent to this dataset. First, all of the data were self-reported and based on surveys that alone are not sufficient to establish psychiatric diagnoses, but rather to identify clinically meaningful symptomatology. Future studies in this area could focus on samples with confirmed clinical diagnoses of hoarding, OCD, and depression. Second, our sample, which was recruited from Amazon’s mTurk mechanism, was predominantly young, female, and highly educated, and therefore not representative of the general population. Our sample also had a much higher than expected proportion of individuals who met clinical cutoffs for hoarding and OCD (even after removing the hoarding-pertinent questions on the OCI-R), consistent with a prior study of mTurk samples that found higher psychopathology in non-clinical samples (Arditte et al., 2016). This perhaps indicates the limitations of self-report or speaks to the unique characteristics of those who tend to use mTurk, and suggests that future studies should include more formal assessments of hoarding and OCD such as the SI-R or Y-BOCS. The high percentages might also be partly attributed to how the study was advertised and worded. It is possible that the phrasing of the advertisement aimed at measuring how one thinks and feels could have attracted more participants with obsessive-compulsive or hoarding symptoms. For example, OCD is characterized by repetitive, intrusive, and anxiety-provoking thoughts while HD is also associated with maladaptive thought patterns (e.g., inability to make a decision on whether to discard an item or fear of making a mistake or error) as well as emotional dysregulation in relation to excessive sentimental attachment toward possessions and distress upon having to discard them. Additionally, our data was collected cross-sectionally at a single timepoint rather than longitudinally, and thus we could not assess the consistency and stability/reliability of responses over time.

Nevertheless, the results of this study further what is currently known of the relationship between hoarding and QoL by examining domain-specific impairments in functioning and accounting for the role of co-morbid depressive symptomatology in a large sample of adults. This work suggests that assessment not only of specific hoarding symptoms, but also of the different components of QoL and any accompanying depression symptomatology could assist therapists and other practitioners in designing treatments aimed at improving the lives and well-being of patients. Future work should address some of the limitations, as well as explore the somewhat unexpected complex relationships between QoL and obsessive–compulsive, hoarding, and depression symptoms.

## Data Availability Statement

The raw data supporting the conclusions of this article will be made available by the authors, without undue reservation.

## Ethics Statement

The studies involving human participants were reviewed and approved by the University of Florida. The patients/participants provided their written informed consent to participate in this study.

## Author Contributions

BN was involved in validating the study, overseeing the project, data curating and conducting of formal analyses, composing the original draft with tables and figures, writing – review and editing, and submitting the manuscript. JZ was involved in conceptualizing and validating the study, formulating the methodology, compiling the study materials and publishing the survey online, data collection and curation, administering and supervising the project, reviewing and editing the manuscript, and providing feedback. LS was involved in validating the study, reviewing and editing the manuscript, and providing feedback. CM was involved in conceptualizing and validating the study, acquiring funding for the project, formulating the methodology, administering and supervising the project, reviewing and editing the manuscript, and providing feedback. All authors contributed to the article and approved the submitted version.

## Conflict of Interest

The authors declare that the research was conducted in the absence of any commercial or financial relationships that could be construed as a potential conflict of interest.

## Publisher’s Note

All claims expressed in this article are solely those of the authors and do not necessarily represent those of their affiliated organizations, or those of the publisher, the editors and the reviewers. Any product that may be evaluated in this article, or claim that may be made by its manufacturer, is not guaranteed or endorsed by the publisher.
