# Supplementary material for: Impact of Hoarding and Obsessive–Compulsive Disorder Symptomatology on Quality of Life and Their Interaction With Depression Symptomatology
Source: Front Psychol. 2022 Aug 1;13:926048. doi: 10.3389/fpsyg.2022.926048 (PMC9376220; doi:10.3389/fpsyg.2022.926048)
Supplement: Supplementary file 1 [file Data_Sheet_1.pdf]

Supplemental Table 1. Summary of QoL studies examining hoarding behavior.

Study	Sample	Quality of Life Measure	Findings
**Studies in primary OCD populations**			
Huppert (2009)	15 history of OCD, 32 current OCD, 19 OCD comorbid with other psychiatric diagnoses	Quality of Life Enjoyment and Satisfaction Questionnaire (QLESQ), Short-Form Health Survey-36 (SF-36)	Hoarding symptom subtype on the OCI-R correlated with impairment on the QLESQ total, social, and family scales and the SF-36 social and work scales
Fontanelle (2010)	53 OCD patients	SF-36	Hoarding, depressive symptoms, and employment status predicted 62% of the variance in social functioning, although depression accounted for most of this variance
Albert (2010)	151 OCD patients	SF-36	Hoarding obsessions/compulsions factor on the YBOCS Symptom Checklist was not significantly related to any SF-36 subscales
Saxena (2011)	34 OCD patients with compulsive hoarding, 137 OCD patients without hoarding	Lehman Quality of Life Interview-Short Form	Hoarding group scored significantly lower on satisfaction with safety and living arrangements and higher on victimization, and trended towards lower scores on satisfaction with living situation; depression scores did not differ between groups
Subramaniam (2014)	175 OCD without hoarding, 122 hoarding without OCD, 55 OCD with hoarding	EuroQol 5 (EQ-5D)	Patients with both OCD and hoarding had a lower mean EQ-5D index (indicating worse QoL) than OCD-only or hoarding-only patients and were more likely to report problems in the mobility and pain domains
Schwartzman (2017)	325 treatment-seeking patients with primary OCD	QLESQ	Hoarding symptoms were associated with decreased household-related QoL, above and beyond OCD and depression severity
**Studies in primary HD populations**			
Ong (2016)	102 HD patients	QLESQ	HD severity was negatively correlated with QoL, but HD severity and QoL were no longer significantly related after controlling for anxiety and depression

Tolin (2019)	54 treatment-seeking HD patients	SF-36	HD group scored significantly lower than age- and sex-matched healthy controls on emotional well-being, role limitations due to emotional problems, vitality, and general health; the SI-R negatively predicted multiple domains of QoL in the whole sample, but was only significant for vitality QoL within the HD group

<u>Supplemental Table 2</u>. Factor loadings and component domains for QoL groupings using factor analysis of the QoLI.

QoLI domain	***QoLI domain components***	Success Factor Loading	Enrichment Factor Loading	Environment Factor Loading	Family Factor Loading
Success	Health	**<u>0.74</u>**	0.07	0.01	-0.10
	Self-Esteem	**<u>0.71</u>**	0.19	-0.03	0.00
	Goals and Values	**<u>0.65</u>**	0.20	-0.03	0.08
	Money	**<u>0.76</u>**	-0.14	0.13	0.04
	Work	**<u>0.63</u>**	-0.02	0.06	0.15
Enrichment	Play	0.36	**<u>0.50</u>**	0.04	-0.05
	Learning	0.21	**<u>0.75</u>**	-0.08	-0.11
	Creativity	-0.01	**<u>0.88</u>**	-0.02	-0.20
	Helping	-0.02	**<u>0.55</u>**	-0.05	0.32
	Friends	-0.08	**<u>0.40</u>**	0.18	0.30
Environment	Home	0.22	-0.03	**<u>0.51</u>**	0.18
	Neighborhood	0.04	-0.08	**<u>0.96</u>**	-0.12
	Community	0.01	0.01	**<u>0.89</u>**	-0.08
Family	Love	0.29	-0.07	-0.12	**<u>0.66</u>**
	Children	0.01	-0.18	-0.10	**<u>0.92</u>**
	Relatives	-0.19	0.25	0.30	**<u>0.41</u>**

Supplementary Table 3. Mean QoL scores (SD in parentheses) by domain and gender.

QoL Domain	Male	Female	t	p
Total	1.53 (1.92)	1.61 (1.85)	-0.98	0.33
Success	1.02 (2.32)	0.88 (2.27)	1.36	0.18
Enrichment	1.58 (1.82)	1.59 (1.95)	-0.18	0.86
Environment	1.35 (2.05)	1.59 (2.15)	-2.58	0.01
Family	1.85 (2.26)	2.38 (2.29)	-5.21	<0.001

Supplemental Table 4. Bivariate Pearson correlations between QoL groupings and psychiatric symptom subscales across the entire sample (n = 2100).

	HRS-SR	OCI-R	PHQ-9	GAD-7
Overall QoL	-0.195	-0.178	-0.568	-0.466
Success QoL	-0.175	-0.153	-0.574	-0.485
Enrichment QoL	-0.142	-0.150	-0.452	-0.382
Environment QoL	-0.145	-0.123	-0.337	-0.273
Family QoL	-0.132	-0.108	-0.360	-0.263
HRS-SR	1	0.601	0.471	0.441
OCI-R	0.601	1	0.515	0.562
PHQ-9	0.471	0.515	1	0.814
GAD-7	0.441	0.562	0.814	1

*All bivariate correlation pairs were significant at the $p < 0.01$ (2-tailed) level.

<u>Supplemental Table 5</u>. Comparison of mean QoL and psychiatric symptomatology between hoarding and non-hoarding subgroups (SD in parentheses), based on a cutoff of 14 on the HRS-SR (controlling for covariates).

	Hoarding (n = 392, 18.7% of sample)	Non-hoarding (n = 1708, 81.3% of sample)	F-value	p-value
Overall QoL	0.97 (1.87)	1.72 (1.85)	38.59	<0.001
Success QoL	0.29 (2.37)	1.09 (2.24)	25.74	<0.001
Enrichment QoL	1.13 (2.03)	1.69 (1.85)	21.27	<0.001
Environment QoL	0.95 (2.09)	1.62 (2.10)	23.68	<0.001
Family QoL	1.68 (2.22)	2.27 (2.30)	17.71	<0.001
HRS-SR	21.05 (5.40)	3.69 (3.89)	5265.04	<0.001
OCI-R	22.86 (12.84)	8.54 (8.05)	717.53	<0.001
PHQ-9	10.73 (6.82)	4.82 (5.19)	325.75	<0.001
GAD-7	9.07 (5.83)	4.22 (4.75)	269.54	<0.001

Supplemental Table 6. Comparison of mean QoL and psychiatric symptomatology between hoarding and depression subgroups (SD in parentheses), based on cutoffs of 14 on the HRS-SR and 10 on the PHQ-9, controlling for covariates.

	Neither (n = 1428)	Hoarding w/o depression (n = 177)	Depression w/o hoarding (n = 280)	Hoarding + depression (n = 215)
Overall QoL	2.12 (1.62)	1.57 (1.54)	-0.34 (1.61)	0.48 (1.98)
Success QoL	1.58 (1.96)	1.02 (1.94)	-1.45 (1.84)	-0.30 (2.53)
Enrichment QoL	2.01 (1.68)[a]	1.65 (1.72)[a]	0.05 (1.82)	0.70 (2.16)
Environment QoL	1.89 (1.99)	1.23 (1.85)[a]	0.23 (2.09)[b]	0.72 (2.24)[ab]
Family QoL	2.59 (2.18)[a]	2.18 (2.13)[a]	0.66 (2.22)	1.27 (2.21)
HRS-SR	3.34 (3.73)	19.25 (4.35)	5.48 (4.23)	22.53 (5.73)
OCI-R	7.43 (7.21)	17.49 (10.59)	14.22 (9.58)	27.28 (12.86)
PHQ-9	2.94 (2.73)	4.54 (3.03)	14.40 (4.09)	15.82 (4.44)
GAD-7	2.89 (3.37)	4.85 (4.22)	10.95 (5.03)	12.54 (4.55)

*All post-hoc pairwise comparisons significantly differed at the $p < 0.05$ level, except the pairs denoted by letters within each outcome category row.

<u>Supplemental Table 7</u>. Comparison of mean QoL and psychiatric symptomatology between hoarding and OCD subgroups (SD in parentheses), based on cutoffs of 14 on the HRS-SR and 21 on the OCI-R, controlling for covariates.

	Neither (n = 1550)	Hoarding w/o OCD (n = 180)	OCD w/o hoarding (n = 158)	Hoarding + OCD (n = 212)
Overall QoL	1.78 (1.83)	0.83 (1.62)[ab]	1.06 (1.97)[ac]	1.09 (2.05)[bc]
Success QoL	1.17 (2.21)	0.04 (1.95)[ab]	0.28 (2.37)[ac]	0.51 (2.66)[bc]
Enrichment QoL	1.76 (1.82)[a]	0.92 (1.85)[bc]	1.00 (2.06)[bd]	1.31 (2.15)[acd]
Environment QoL	1.67 (2.07)	0.88 (2.01)[ab]	1.07 (2.32)[ac]	1.01 (2.15)[bc]
Family QoL	2.30 (2.29)[ab]	1.66 (2.29)[cd]	1.96 (2.39)[ace]	1.70 (2.16)[bde]
HRS-SR	3.55 (3.82)	18.99 (4.61)	5.11 (4.32)	22.80 (5.42)
OCI-R	6.64 (5.29)	11.16 (5.23)	27.15 (6.62)	32.79 (8.15)
PHQ-9	4.41 (4.84)	8.36 (6.18)[a]	8.86 (6.63)[a]	12.74 (6.71)
GAD-7	3.75 (4.36)	7.07 (5.40)	8.77 (5.83)	10.76 (5.65)

*All post-hoc pairwise comparisons significantly differed at the $p < 0.05$ level, except the pairs denoted by letters within each outcome category row.

<u>Supplemental Table 8</u>. Multiple linear regression including the HRS-SR as a predictor of QoL in depressed and non-depressed groups based on a PHQ-9 cutoff of 10. Each overall model (including covariates) was statistically significant ($p < 0.05$). Unstandardized beta coefficients are given in parentheses.

QoL Domain	HRS-SR Beta Coefficient (Standardized, Unstandardized) (depressed group, n = 495)	t	p-value	Adjusted R-Square, F-value	HRS-SR Beta Coefficient (Standardized, Unstandardized) (non-depressed group, n = 1605)	t	p-value	Adjusted R-Square, F-value
Overall	0.252 (0.047)	5.93	<0.001	0.148, 11.767	-0.149 (-0.038)	-6.17	<0.001	0.087, 20.057
Success	0.260 (0.059)	6.14	<0.001	0.156, 12.413	-0.115 (-0.036)	-4.76	<0.001	0.087, 20.091
Enrichment	0.194 (0.040)	4.33	<0.001	0.052, 4.407	-0.098 (-0.026)	-3.91	<0.001	0.018, 4.628
Environment	0.135 (0.030)	3.01	0.003	0.057, 4.721	-0.127 (-0.040)	-5.21	<0.001	0.060, 13.791
Family	0.167 (0.038)	3.85	<0.001	0.119, 9.373	-0.098 (-0.034)	-4.28	<0.001	0.180, 44.953

<u>Supplemental Table 9.</u> Multiple linear regression comparing the HRS-SR and OCI-R as predictors of home, neighborhood, community, love, children, and relatives QoL subcomponents in separate models (covariates not shown). Unstandardized beta coefficients are given in parentheses.

QoL Subcomponent	OCI-R Beta Coefficient (Standardized, Unstandardized)	t	p	Adjusted R-Square, F-value	HRS-SR Beta Coefficient (Standardized, Unstandardized)	t	p	Adjusted R-Square, F-value
Home	-0.101 (-0.028)	-4.63	<0.001	0.062, 18.22	-0.153 (-0.057)	-7.16	<0.001	0.075, 22.16
Neighborhood	-0.08 (-0.018)	-3.65	<0.001	0.036, 10.69	-0.094 (-0.029)	-4.34	<0.001	0.038, 11.41
Community	-0.072 (-0.015)	-3.28	0.001	0.033, 9.84	-0.047 (-0.013)	-2.14	0.03	0.030, 9.05
Love	-0.076 (-0.026)	-3.72	<0.001	0.177, 57.46	-0.088 (-0.040)	-4.40	<0.001	0.179, 58.28
Children	-0.039 (-0.011)	-1.85	0.07	0.128, 39.35	-0.052 (-0.020)	-2.52	0.01	0.129, 39.77
Relatives	-0.092 (-0.023)	-4.14	<0.001	0.012, 4.28	-0.109 (-0.036)	-4.94	<0.001	0.016, 5.19

<u>Supplemental Table 10.</u> Multiple linear regression including both the HRS-SR and OCI-R in the same model as predictors of home, neighborhood, community, love, children, and relatives QoL subcomponents (covariates not shown). Unstandardized beta coefficients are given in parentheses.

QoL Subcomponent	OCI-R Beta Coefficient (Standardized, Unstandardized)	t	p	HRS-SR Beta Coefficient (Standardized, Unstandardized)	t	p	Adjusted R-Square, F-value
Home	-0.015 (-0.004)	-0.58	0.56	-0.144 (-0.054)	-5.46	<0.001	0.074, 19.73
Neighborhood	-0.037 (-0.008)	-1.37	0.17	-0.073 (-0.022)	-2.71	0.007	0.039, 10.35
Community	-0.068 (-0.014)	-2.49	0.01	-0.007 (-0.002)	-0.28	0.78	0.032, 8.76
Love	-0.036 (-0.012)	-1.42	0.16	-0.068 (-0.031)	-2.73	0.006	0.180, 52.06
Children	-0.012 (-0.003)	-0.46	0.64	-0.045 (-0.018)	-1.77	0.08	0.128, 35.36
Relatives	-0.043 (-0.011)	-1.55	0.12	-0.084 (-0.028)	-3.09	0.002	0.016, 4.88

<u>Supplementary Table 11</u>. Multiple linear regression including both the HRS-SR and OCI-R in the same model, with the addition of the PHQ-9, as predictors of home, neighborhood, community, love, children, and relatives QoL subcomponents (covariates not shown). Unstandardized beta coefficients are given in parentheses.

QoL Subcomponent	OCI-R Beta Coefficient (Standardized, Unstandardized)	t	p	HRS-SR Beta Coefficient (Standardized, Unstandardized)	t	p	PHQ-9 Beta Coefficient (Standardized, Unstandardized)	t	p	Adjusted R-Square, F-value
Home	0.112 (0.031)	4.17	<0.001	-0.056 (-0.021)	-2.15	0.03	-0.361 (-0.180)	-14.61	<0.001	0.160, 40.90
Neighborhood	0.047 (0.011)	1.66	0.10	-0.015 (-0.004)	-0.54	0.59	-0.238 (-0.096)	-9.19	<0.001	0.075, 18.12
Community	0.010 (0.002)	0.37	0.72	0.047 (0.013)	1.72	0.09	-0.222 (-0.084)	-8.50	<0.001	0.064, 15.37
Love	0.075 (0.025)	2.96	0.003	0.009 (0.004)	0.37	0.71	-0.314 (-0.190)	-13.39	<0.001	0.244, 68.79
Children	0.062 (0.018)	2.31	0.02	0.006 (0.002)	0.24	0.81	-0.210 (-0.108)	-8.47	<0.001	0.157, 40.07
Relatives	0.067 (0.017)	2.40	0.02	-0.008 (-0.003)	-0.29	0.77	-0.312 (-0.139)	-12.04	<0.001	0.080, 19.18
